# Plaque heterogeneity and the spatial distributions of its components dictate drug-coated balloon therapy

**DOI:** 10.1038/s41598-024-54756-9

**Published:** 2024-02-22

**Authors:** Sarifuddin, Prashanta Kumar Mandal

**Affiliations:** 1 Department of Mathematics, Berhampore College, Berhampore, Murshidabad, W.B. 742 101 India; 2 Department of Mathematics, Visva-Bharati, Santiniketan, W.B. 731 235 India

**Keywords:** Drug-coated balloon, Plaque heterogeneity, Spatial distributions, MAC and IBM method, Tissue content, Applied mathematics, Biophysics, Computational biology and bioinformatics, Translational research

## Abstract

Drug-coated balloon (DCB) angioplasty is one of the potential approaches to alleviating in-stent restenosis and treating peripheral artery disease. An in-silico model has been developed for sirolimus drug eluted from an inflated balloon in a patient-specific arterial cross-section consisting of fibrous tissue, fibrofatty tissue, dense calcium, necrotic core, and healthy tissue. The convection-diffusion-reaction equation represents the transport of drug, while drug binding, both specific and non-specific, can be modelled as a reaction process. The Brinkman equations describe the interstitial flow in porous tissue. An image processing technique is leveraged for reconstructing the computational domain. The Marker and Cell, and Immersed Boundary Methods are used to solve the set of governing equations. The no-flux interface condition and convection do amplify the tissue content, and the regions of dense calcium and necrotic core limited to or extremely close to the interface pose a clinical threat to DCB therapy. Simulations predict the effects of the positioning and clustering of plaque components in the domain. This study demands extensive intravascular ultrasound-derived virtual histology (VH-IVUS) imaging to understand the plaque morphology and determine the relative positions of different plaque compositions about the lumen-tissue interface, which have a significant impact on arterial pharmacokinetics.

## Introduction

High rates of in-stent restenosis (ISR) have driven increasing interest in endovascular drug delivery using drug-coated balloons (DCBs), containing an antiproliferative drug to inhibit intimal hyperplasia (IH) caused by abnormal growth of smooth muscle cells (SMCs) present in *tunica media*^1–5^. DCBs have revolutionised the treatment of restenosis at the sites of grafting or ISR following stent deployment or de novo lesion. Although, DCB is still a promising technology, with no other options open, there are still limitations to DCB therapy because of significant drug loss ($\sim$ 80–90%) en route to the target lesion or downstream during the procedure and the drug’s inability or weak ability to permeate into a severely calcified lesion^6–8^. Most studies using DCB have used paclitaxel (PTX) and have demonstrated varying levels of efficacy^9,10^. In response to a meta-analysis of randomised controlled trials of PTX-coated balloons demonstrating excess late mortality starting after 2 years^11^, the US Food and Drug Administration (FDA) advised interventionists to use DCB in only the highest-risk patients, followed by careful follow-up. On the basis of its underlying assumptions, some experts questioned the validity of this meta-analysis and indicated that PTX-based DCBs might really be safe^12^. The causal mechanism connecting PTX with excessive late mortality is still unknown, but it has focused our understanding of how the DCB device functions^13^. Therefore, there is an urgent need for a greater understanding of the variables that control this endovascular therapy’s effectiveness and limit its negative systemic consequences^14,15^.

There are a plethora of experimental and/or computational studies on endovascular drug delivery in the literature, but studies on DCB delivery are scarce to date. In hypercholesterolemic femoral arteries of swine, the efficacy of zotarolimus-coated balloon delivery is estimated^16^. In another study, an integrated approach combining animal studies, bench-top experiments and computational modelling showed zotarolimus concentration decreases with transmural depth as opposed to multiple peaks displayed by PTX^17^. Some researchers^18^ studied drug-coated balloon and drug-eluting stent therapies using physics-based computational modelling and data-driven machine learning to predict high-fidelity solutions on refined meshes by producing data from low-fidelity coarse meshes. Because arterial ultrastructure and lesion complexity dictate tissue content in the case of endovascular delivery^19–22^, quantifying arterial pharmacokinetics in a patient-specific heterogeneous plaque composition is critical to understanding DCB delivery. Furthermore, the presence of calcium in a plaque acts as an impenetrable structural diffusion barrier that prevents tissue from being accessed. To overcome this problem, a study on intraluminal PTX delivery to human peripheral arteries with significant calcified plaque estimates that calcium removal increases diffusivity by 70%^23^ following orbital atherectomy. In another study^24^, the hindered diffusivity in the calcified region measures 10- and 100-fold lower compared to the healthy vessel. Furthermore, some studies demonstrate the strong dependence of tissue drug concentration on arterial ultrastructure and provide a rational basis by which to understand real-world scenarios, and conclude that the local tissue composition needs to be carefully characterised in the context of modelling DCB therapy^25–29^.

Primary patency rates are still unsatisfactory despite the benefits of DCB delivery because of a number of understudied issues, such as coating-tissue bond failure (adhesive or cohesive)^30^. Double balloons or different excipients are also used to modify balloon structures for improved local delivery^31^. The fundamentals of drug uptake from an inflated DCB into an interface are based on multiple modes of interaction. The surface topography, the coating morphology, and the balloon contact pressure modulate acute drug transfer during deployment^32^. It has been demonstrated that a linearly micro-patterned drug-eluting balloon does improve the efficacy as well as the accuracy of DCB delivery^33^, and hence a firm contact between the surface of an inflated balloon and the lumen-tissue interface is an essential requirement for drug uptake. Again, the unique microstructures of the coating, having specific contact pressure, can impact acute drug transfer by considering urea and shellac as drug carriers^34^. Some studies on (i) surface modification using ultraviolet-ozone plasma treatment to increase the hydrophilicity of the balloon surface^35^, (ii) use of microneedle drug-eluting balloon in an atherosclerosis rabbit model^36^, aim to enhance drug delivery from an inflated balloon and to get better retention. The recent article^37^ does estimate the loss of drug/excipient during angioplasty for various coating technologies of DCB. The dependence of boundary conditions in inner and outer surfaces on balloon-based delivery of an anti-proliferative drug in a multi-layered vessel wall has been successfully carried out^15^. In a recent study^38^, the retention of drug for various excipients (urea and butyryl-trihexyl citrate (BTHC)), where just the inner-most layer (intima) was made to mimic an atheroma in diseased conditions, has been predicted in an idealised atherosclerotic vessel by estimating that urea, despite having a higher tracking loss than BTHC, is linked to substantially greater and faster drug absorption than BTHC. In the case of DCB delivery, the majority of the research mentioned above took into account an idealised artery. As the therapeutic domain is an atherosclerotic artery, and the presence of atherosclerotic plaque with heterogeneous tissue compositions influencing vascular pharmacokinetics is likely to become a critical consideration for the success of an intravascular delivery device, modelling a realistic atherosclerotic artery will undoubtedly have the potential to estimate the tissue content in a heterogeneous atherosclerotic artery. A seminal article by some assiduous researchers pointed out that in atherosclerotic vasculature, with no specific assessment of lesion morphology, a paradoxical overriding aspect dictates retention and response^39^. Therefore, one of the elements needed to improve interventional success is a shift to lesion-specific intervention, where the lesion phenotype and vascular characteristics predominantly dictate the therapy^40^.

Although PTX is commonly coated on DCBs for clinical usage, sirolimus-eluting balloons have come into focus due to the potential mortality signal provided by PTX-coated balloons in peripheral procedures. While the importance of downstream embolic consequences following DCB usage is yet unknown, a number of preclinical investigations indicate that these side effects may be hazardous. High drug-tissue levels are made possible by hydrophilic excipient coatings of PTX, but they also facilitate high coating wash-off prior to delivery to the target site^41^. It is possible for the drug/excipient to become embolised downstream when PTX is lost into the body, even if it is not at the target site. It is unsure if this could lead to adverse results, although it is an undesirable effect of the PTX DCB technologies that are in use today. The necessity of sirolimus DCBs has been highlighted by recent safety concerns regarding PTX DCBs^11^.

Enhancing the drug coating’s robustness to reduce drug loss during tracking, enable smoother transfer to the vessel wall, and enhance drug uptake without generating embolisation downstream are the main goals of research and development efforts for the more recent generation of DCB. The efficacy of limus-analogues, such as sirolimus, sometimes referred to as rapamycin, can be affected by their rapid diffusion into surrounding tissue in the absence of a protective layer^42^. Numerous trials have demonstrated the safety and effectiveness of sirolimus DCB in coronary artery interventions^43,44^. Theoretically, sirolimus has a wider therapeutic range and an advantage over PTX due to its anti-inflammatory and anti-restenosis effects^45^. Sirolimus may help to mitigate any cytotoxicity of PTX, but more clinical studies are needed to ascertain whether sirolimus DCB will eventually replace PTX DCB.

In this investigation, we take into account a cross-sectional image of a patient-specific atherosclerotic artery with heterogeneous tissue compositions comprising healthy tissue (‘HT’), and regions of fibrous tissue (‘FI’), fibrofatty tissue (‘FF’), dense calcium (‘DC’) and necrotic core (‘NC’) obtained from VH-IVUS^46^. An image processing technique based on an unsupervised *k*-means clustering algorithm is leveraged to automatically detect segmentations based on colours^47^. It is widely known that when drug molecules bind to specific receptors (SRs), there is also an occurrence of non-specific binding caused by the trapping of drug in the extracellular matrix (ECM)^48–54^. Since arterial ultrastructure modulates drug delivery from coated balloons, an attempt is made to investigate the DCB delivery in a patient-specific atherosclerotic artery, which is driven by the two-phase binding of drug (specific and non-specific) by applying a nonlinear reversible chemical reaction process in the tissue. To this aim, the transport of free drug is modelled as a convection-diffusion-reaction process, while the specific and non-specific binding of drug is modelled as a reaction process only. The Marker and Cell (MAC)^55^, and Immersed Boundary Method (IBM)^56,57^ are leveraged to gain a quantitative understanding of the model considered, where each pixel of the cross-sectional image is regarded as a control volume. The effect of convection has been estimated by taking into consideration the flow of interstitial fluid in a diseased porous wall. As a precise lumen-tissue interface condition for free sirolimus is not apparently available, this model does account for two opposing extremes, namely, no-flux and wash-out (sink) situations at the interface after the deflation of the balloon catheter. The assumption of no-flux and wash-out conditions at the interface is a crude approximation as it does not resemble the real situation. This study incorporates additional considerations of plaque refinement by looking at (i) healthy only (Healthy model), (ii) HT + DC and NC (Hard model), and (iii) HT + FI and FF (Soft model) in order to assess various pharmacological factors that pose a clinical threat to interventional cardiologists. In a bid to validate our findings, a circular geometry in the line of^58^ has also been considered.

In contrast to earlier studies on DCB therapy, this study’s innovative aspects include the inclusion of a patient-specific artery cross-section as a therapeutic domain and the two-phase binding of the drug in a heterogeneous plaque. Since, one of our objectives is to study the impact of positional variations of each plaque component in a non-clustered form on endovascular delivery using DCB, we have reconstructed a number of model geometries from the Ref. model (cf. Types- I, II, III, and IV; Table 3). We also recreated another set of model geometries (cf. Types- A, A1, A2, A3, B, and C; Table 4) based on the clustered position of DC and NC regions about the interface. The investigation’s novel feature focuses on the spatially diverse tissue compositions and how they affect DCB therapy. Significant impacts of these factors on drug distribution as well as retention are noted in this investigation. Unlike healthy vessels, the consideration of varying plaque compositions gives rise to non-uniform drug distribution as well as retention in the therapeutic domain. Due to the uncertainty of some parameters involved, a thorough sensitivity analysis has been carried out. As far as the authors are aware, there is no research that takes arterial pharmacokinetics into consideration based on these crucial factors. Last but not least, this study demands extensive VH-IVUS imaging to understand the plaque morphology and to determine whether it requires treatment prior to intervention, specifically when the regions of DC and NC are limited to or very close to the interface. Therefore, in order to improve the efficacy of DCB therapy, the notion that ‘one size fits all’ needs to be revisited.

## Materials and methods

### Boundary detection

Using our in-house developed Matlab code, the inner (lumen-tissue) and outer (perivascular) boundaries of the cross-sectional geometry have been detected by extracting a set of connected points. A search algorithm has been leveraged to identify the points that lie outside or inside the domain of computation (tissue). The location of a grid point (i.e. inside or outside) with respect to the boundaries is determined from the dot product of the surface normal vector ($\mathbf{n_i; i=I,A}$) and the position vector (**p**) of the grid point drawn from the boundary. The grid point is identified as being in the tissue domain or outside the tissue domain according to $\mathbf{p.n_i, \;(i=I,A)} \le 0$ or $\mathbf{p.n_i, \;(i=I,A)} > 0$ respectively.

### Geometry reconstruction

**Figure 1:**
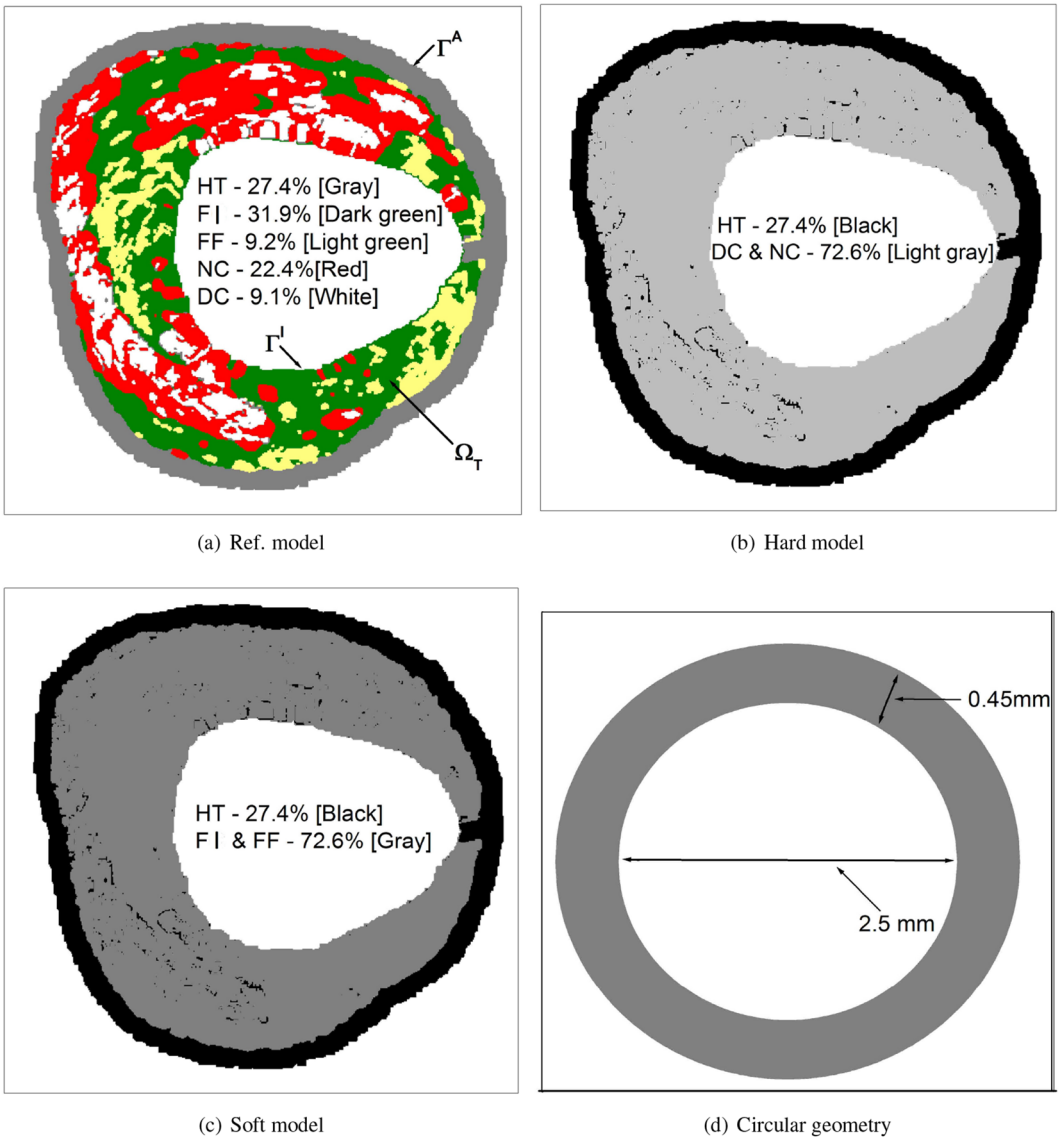
Geometry and computational domain with heterogeneous tissue compositions: (**a**) Ref. model; (**b**) Hard model; (**c**) Soft model; (**d**) Circular cross-section.

Automatic characterisation of an atherosclerotic plaque consisting of heterogeneous compositions derived from VH-IVUS has been considered. The borderlines are detected by an automatic boundary detection algorithm in Matlab code. Image segmentation has been performed on this cross-sectional arterial vessel using an unsupervised *k*-means clustering algorithm based on colours to detect different sub-domains of the arterial vessel (cf. Fig. 1a). To study the effects of different plaque compositions, the three different models of the atherosclerotic arterial cross-section in Fig. 1a–c have been examined. The first shape considered here is the patient-specific asymmetric model, which mimics real surface irregularities since the actual variation of the cross-sectional area is retained (termed as a ‘Reference (Ref.) model’; Fig 1a). The Ref. model consists of healthy tissue (‘HT’) (gray: 27.4%), fibrous tissue (‘FI’) (dark green: 31.9%), fibrofatty tissue (‘FF’) (light green: 9.2%), necrotic core (‘NC’)(red: 22.4%) and dense calcium (‘DC’)(white: 9.1%). The second geometrical model considered consists of HT (black: 27.4%) and DC and NC (light gray: 72.6%) (termed as a ‘Hard model’, Fig. 1b). Finally, by introducing FI and FF in place of DC and NC in ‘Hard model’, we get ‘Soft model’ which has HT (black: 27.4%) and FI and FF (gray: 72.6%) (cf. Fig. 1c). In a bid to make a comparative study with that of a healthy vessel, the present investigation also deals with a ‘Healthy’ model consisting of HT only (100%). Furthermore, in order to validate the applicability of our model as well as our findings, a circular geometry in the line of^58^ is used in this investigation (cf. Fig. 1d). Concomitantly, simulations on a number of reconstructed geometries (cf. Tables 3, 4) clearly highlight the influence of spatial variation of each plaque component dispersed in a non-clustered or clustered manner on drug distribution and retention in varying tissue compositions.

**Table 1:** Models used to study various plaque compositions.

Model	HT (%)	FI (%)	FF (%)	NC (%)	DC (%)
Ref. model	27.4	31.9	9.2	22.4	9.1
Hard model	27.4	–	–	72.6	
Soft model	27.4	72.6		–	–
Healthy model	100	–	–	-	–

### Equations for interstitial flow through porous tissue

The governing equations representing the interstitial flow in the porous tissue are the Brinkman equations and the equation of continuity, whose respective forms are as follows:

1 $$\begin{aligned} \rho _t \Big \lbrace {\partial \textbf{w} \over \partial t}+ (\mathbf{w \cdot } \nabla )\textbf{w} \Big \rbrace= & {} -{\nabla P} + {\nabla \mathbf{\cdot } \tau } -\frac{\mu _t}{K}{} \textbf{w}, \end{aligned}$$

2 $$\begin{aligned} \nabla \mathbf{\cdot w}= & {} 0, \;\;\;\;\;\;\;\;\;\; \textrm{in} \;\;\; { {\Omega }_T},\nonumber \\ \textrm{where }\;\;\; \tau= & {} {\mu _t}\Big \lbrace \nabla \textbf{w}+(\nabla \textbf{w})^T\Big \rbrace . \end{aligned}$$

Here, $\textbf{w}$ is the interstitial velocity-vector in the tissue, $\rho _t$, $\mu _t$, *P*, $\tau$, *K* are the density, the viscosity, the pressure, the stress tensor and the Darcy permeability respectively.

### Boundary conditions for interstitial flow

Since the lumen-tissue interface ($\Gamma ^I$) is not regular, multi-directional transmural plasma filtration from the lumen into tissue takes place. Due to non-availability of data, an all-time equal prescribed filtration velocity (*V*)^59^ along the interface has been assumed. Therefore, mathematically, we have

3 $$\begin{aligned} \mathbf{w \cdot n_I} = V, \; \textrm{on}\; \Gamma ^I, \end{aligned}$$

where $\mathbf{n_I}$ is the unit outward normal vector on $\Gamma ^I.$

At the adventitial end ($\Gamma ^A$), zero variation for the velocity in normal direction is assumed which may be written mathematically as^60^

4 $$\begin{aligned} \frac{\partial \textbf{w}}{\partial \mathbf{n_A}} = 0\; \textrm{on} \; \Gamma ^A, \end{aligned}$$

where $\mathbf{n_A}$ is the unit outward normal vector on $\Gamma ^A.$

### Equations for free and two-phase bound drug

The volume-averaged molar concentration of free drug in the tissue eluted from a DCB is denoted by $c_t.$ The free drug is allowed to reversibly bind to its specific receptors and extracellular binding sites to get the volume-averaged concentrations of REC ($b_R$)- and ECM ($b_E$)-bound drug respectively. The governing equations representing the dynamics of the free, the REC- and the ECM-bound drug are given by

5 $$\begin{aligned} {\partial c_t \over \partial t}+ (\mathbf{w \cdot } \nabla ) c_t= & {} D_t^l {\nabla ^2} c_t - \frac{\partial b_{R}}{\partial t} - \frac{\partial b_E}{\partial t}, \end{aligned}$$

6 $$\begin{aligned} {\partial b_{R} \over \partial t}= & {} k_{Ra}(B_{Rm} - b_{R})c_t - k_{Rd}b_{R}, \end{aligned}$$

7 $$\begin{aligned} {\partial b_{E} \over \partial t}= & {} k_{Ea}(B_{Em} - b_{E})c_t - k_{Ed}b_{E}, \end{aligned}$$

where $k_{Ra}$, $B_{Rm}$ and $k_{Rd}$ are the association rate constant, the maximum tissue binding capacity and the dissociation rate constant respectively for the REC-bound drug and the corresponding quantities $k_{Ea}$, $B_{Em}$ and $k_{Ed}$ are for the ECM-bound drug. Here, $D_t^{l}\; (l=1,2,3,4,5)$ are the diffusion coefficients for the free drug in the diseased atherosclerotic vessel for the different types of plaque compositions, namely, fibrous tissue, fibrofatty tissue, necrotic core, calcified lesion and healthy tissue respectively. In the line of previous studies, equal diffusivities for FI and FF, and DC and NC are assumed due to nonavailability of data.

### Interface and boundary conditions for drug

The kinetic equation for the release of sirolimus from the balloon may be taken as^58^

8 $$\begin{aligned} M_b(t) = a_1(1- e ^{-k_1 t}); t \le t_0, \end{aligned}$$

where $k_1$ and $a_1$ are empirical constants and $t_0$ is the balloon inflation time. It may be noted that the same empirical relationship was used earlier in the case of zotarolimus release from a DCB^17^.

Once the balloon is deflated and retracted from the arterial lumen, the bulk of the transferred drug at the lumen-tissue interface becomes exposed to the streaming blood. Since a proper boundary condition at the interface is not readily apparent, we assume two extremes in this model by considering the luminal flow is insensitive to the flowing blood (treated as a no-flux condition) or the luminal flow is efficient enough to syphon out the mural-adhered drug (treated as a sink condition)^26^. As a result, after deflation of the balloon ($t > t_0$), the following boundary conditions may be used at the lumen-tissue interface ($\Gamma ^I$):

9 $$\begin{aligned} J_I = 0 \; \textrm{or} \; c_t=0 \; \textrm{on}\; \Gamma ^I,\; t >t_0, \end{aligned}$$

where $J_I$ represents the interfacial flux for the free drug.

At the adventitial end ($\Gamma ^A$), a perfectly sink condition may be mathematically written as^61^

10 $$\begin{aligned} c_t=0 \; \textrm{on} \; \Gamma ^A. \end{aligned}$$

The initial conditions for the free and bound drug are assumed to be zero.

### Determination of tissue content and fractional effects

We now determine the tissue content [$C^{\rm tissue}(t),$ (g drug /g tissue)] for the tissue domain as

11 $$\begin{aligned} C^{\rm tissue}(t) = \frac{D_{MW}}{{\rho _{t}} A} \underset{\Omega _T}{\iint }\ \big [{c_t +b_R + b_E}\big ]dA, \end{aligned}$$

where $D_{MW}$ is the molecular weight of the drug and *A* is the area of the tissue domain.

The fractional effect of REC-bound drug [$\textrm{FE}^R(t)$] in the tissue domain may be estimated from

12 $$\begin{aligned} \textrm{FE}^R (t) = \frac{1}{A} \underset{\Omega _T}{\iint }\ \Big [{b_R \over {B_{Rm}}}\Big ]dA. \end{aligned}$$

By the same token, the fractional effect of ECM-bound drug [$\textrm{FE}^E (t)$] in the therapeutic domain may be written as

13 $$\begin{aligned} \textrm{FE}^E(t) = \frac{1}{A} \underset{\Omega _T}{\iint }\ \Big [{b_E \over {B_{Em}}}\Big ]dA. \end{aligned}$$

### Computational procedures

#### Marker and cell (MAC) methodology

**Figure 2:**
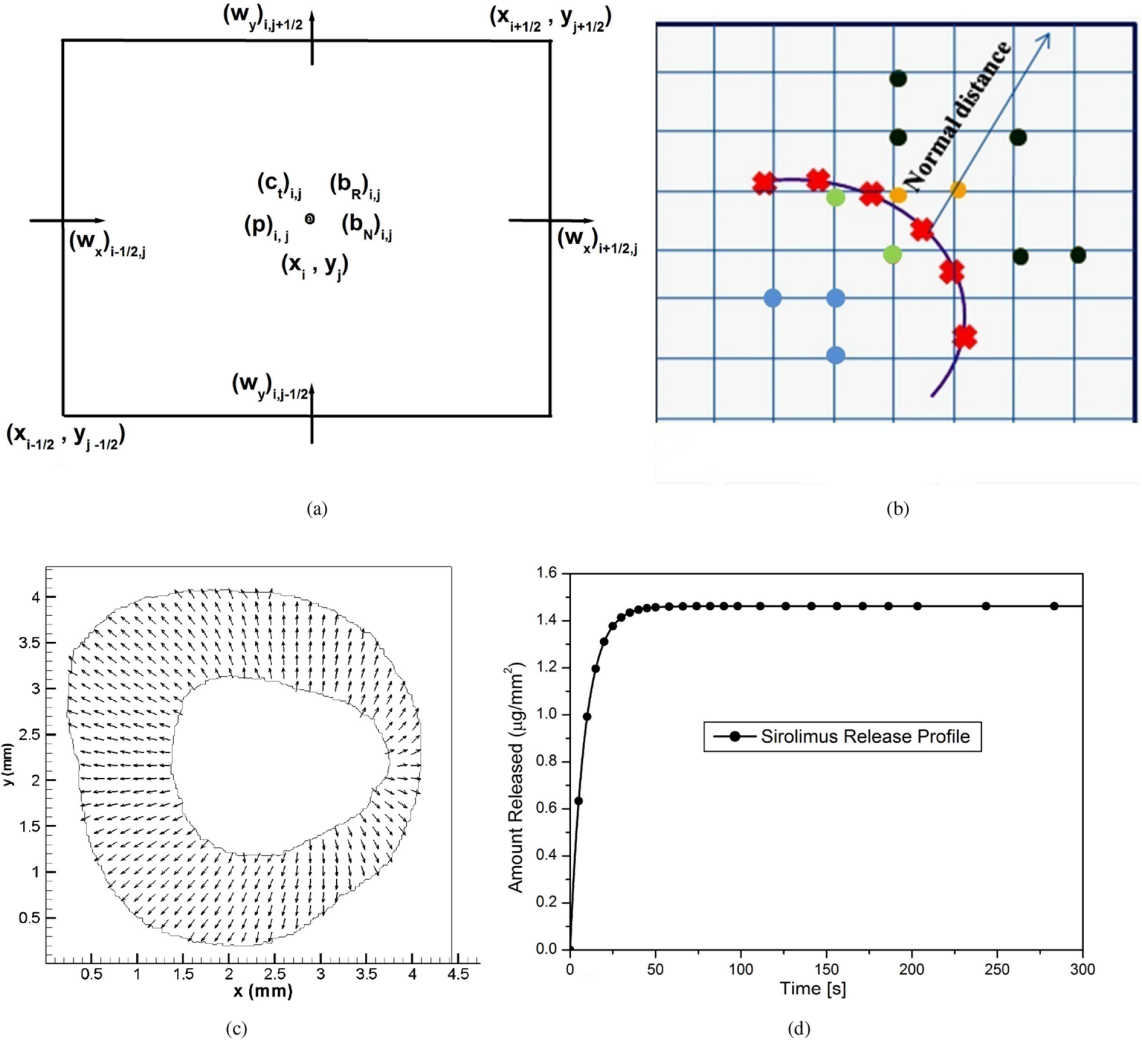
(**a**) A typical MAC cell; (**b**) IBM nodes demarcation and interpolation; (**c**) Interstitial velocity-vector (not to scale); (**d**) Sirolimus release profile during balloon angioplasty.

While reconstructing the geometry, an image segmentation procedure based on five different colours has been adopted to get the non-homogeneous, irregular computational domain consisting of 119329 pixels. For computational purposes, each pixel is considered as a control volume. Control volume-based finite-difference discretisation of the governing equations and boundary conditions is carried out in staggered grids, usually known as the MAC method. In this type of grid alignment, the interstitial velocity components, the pressure, and the drug concentrations are calculated at different locations of the control volume (cf. Fig. 2a). The discretisation of the time derivative terms is based on the first-order accurate two-level forward time differencing formula, while the diffusive terms are discretised by the second-order accurate three-point central difference formula. The convective terms for interstitial flow are discretised with a hybrid formula consisting of central differencing and a second-order unwinding, while that appearing in the drug transport equation is discretised by non-oscillatory scheme^62^. The equation for pressure, derived from the discretised continuity and momentum equations, is solved iteratively by the Successive-over-Relaxation (SOR) method with the chosen value of the over-relaxation parameter as 1.2 in order to get the intermediate pressure-field using the interstitial velocity-field. Subsequently, the maximum cell-divergence of the velocity-field is calculated and checked for its tolerance. If the tolerance limit ($10^{-12}$) is not satisfied, then the pressure at each cell of the domain is corrected, and the interstitial velocities at each cell are adjusted accordingly by the SOLA scheme^63^.

#### Immersed boundary method (IBM)

The immersed boundary method has been leveraged to tackle the irregular domain boundaries in the background of a Cartesian mesh. A direct forcing approach has been used in the present implementation. The curved interface is described using a set of connected points (cf. Fig. 2b). The boundary conditions for the interstitial fluid, the free and bound drug at the inner and outer boundaries are imposed explicitly at the beginning of each time-iteration. For the sake of brevity, the details are not given here. However, interested readers may refer to^64^ for further details.

#### Stability criteria

By using an adaptive time stepping routine, which automatically selects the time step most suitable for the velocity-field at that cycle, the number of computation cycles and, thus, the running time, could be decreased^65^. In this investigation, three distinct stability criteria have been used: two for interstitial fluid and one for free drug transport, which are stated as

(i) Time step $(\delta {t_1})$ due to involvement of Reynolds number^66,67^.
(ii) Time step $(\delta {t_2})$ due to the movement of the fluid particles^68^.
(iii) Time step $(\delta {t_3})$ depending on the diffusivity as well as the dimensions of the control volume (Courant–Friedrichs–Lewy (CFL) stability)^69^.

Finally, the time step ($\delta t)$ chosen for the simulation purpose is the minimum of the time steps calculated by the above three stability criteria. For the sake of brevity, details of these time-stepping formulations are not given here. However, interested readers are referred to^27^ for further details.

#### IBM-MAC algorithm

The algorithm for the proposed IBM-MAC methodology can be summarised as follows:

- To identify the cells that are intercepted, i.e., cut-cells (cells where some grid points fall inside as well as outside the tissue).
- To identify the nodes in each cut-cell for the interstitial velocities, the pressure, the free drug, and the REC- and ECM-bound drug concentrations in the tissue.
- To initialise the velocity components and concentrations for each cell in the tissue. This is done either from the result of the previous cycle or from the prescribed initial conditions.
- To calculate the time step ($\delta t$) calculated from the stability criteria (cf. Section “Computational procedures”).
- To apply the MAC and SOR methods for solving the momentum and pressure equations respectively.
- To interpolate/extrapolate variables at the cut-cell nodes along a surface normal direction using the geometrical location of the boundary and neighboring nodes.
- To calculate the maximum cell divergence and check for its tolerance. If the maximum divergence of the interstitial velocity-field is found to be greater than the tolerance limit at any cell in an absolute sense, then apply pressure-velocity correction technique in regular cell and cut-cell (as per SOLA) until maximum cell divergence is satisfied with the desire degree of accuracy in an iterative manner.
- To solve the transport equations for drugs to get concentrations within the arterial tissue in an explicit manner provided the tolerance limit is satisfied.

This completes the necessary calculations for advancing flow-field and drug concentration through one cycle at a time. Our in-house developed FORTRAN code based on this algorithm has been used for computational purposes.

## Results

A patient-specific artery cross-section with diverse plaque compositions has been used as the computational domain for the purpose of numerical computation of the desired quantities of important physiological significance. Lesion-specific transport properties (as exemplified by the diffusion coefficient) along with the spatial positions of individual plaque components and their appearances do modulate drug distribution as well as retention in the atherosclerotic domain. Solutions in this computational domain with 119329 pixels are computed by pixel-based generation of staggered grids in which the area of each pixel is $7.19 \times 10^{-7}$ cm^2^. The plausible values of input parameters are given in Table 2. Steady states have been achieved when there was a $10^{-6}$ reduction in the drug transport residual and the divergence of velocity-field is less than $10^{-12}$ at any cell in the absolute sense. Each run to achieve the steady state for the Ref. model takes approximately 5.46 h in Ubuntu 20.04/Linux OS using AMD Fx(tm)-6300, a six-core processor   3.5 GHz, and 4 GB RAM.

**Table 2:** Parameters used in the computational model for sirolimus drug.

Description	Parameters	Values	References
Tissue density (g/ml )	$\rho _{t}$	0.983	^70^
Viscosity (g/(mm s))	$\mu _t$	$5\times 10^{-3}$	^22^
Permeability (mm^2^)	*K*	$1.43\times 10^{-12}$	^71^
Filtration velocity (mm/s)	*V*	$5.8 \times 10^{-5}$	^59^
Diffusion coefficients $(D_{t}^{l}$ $(mm^2 /s)$			
fibrous tissue	$D_{t}^{1}$	$1.0 \times 10^{-4}$	^38^
fibrofatty tissue	$D_{t}^{2}$	$1.0 \times 10^{-4}$	^38^
Necrotic core	$D_{t}^{3}$	$8.42 \times 10^{-7}$	Estimated
Calcified lesion	$D_{t}^{4}$	$8.42 \times 10^{-7}$	Estimated
Healthy tissue	$D_{t}^{5}$	$8.42 \times 10^{-5}$	^38^
Maximum receptor-binding capacity (mol/mm^3^)	$B_{Rm}$	$3.3 \times 10^{-12}$	^70^
Maximum ECM-binding capacity (mol/mm^3^)	$B_{Em}$	$3.6 \times 10^{-10}$	^70^
Receptor binding-on rate (mol mm^-3^ s)^-1^	$k_{Ra}$	$8.0\times 10^{11}$	^70^
ECM binding-on rate (mol mm^-3^ s)^-1^	$k_{Ea}$	$2.0\times 10^{9}$	^70^
Equilibrium dissociation rate (receptor) (s^-1^)	$k_{Rd}$	$1.6 \times 10^{-4}$	^70^
Equilibrium dissociation rate (ECM) (s^-1^)	$k_{Ed}$	$5.2 \times 10^{-3}$	^70^
Empirical constant (s^-1^	$k_1$	0.1135	^58^
Empirical constant ($\mu$g mm^-2^)	$a_1$	1.4618	^58^
Balloon inflation time (*s*)	$t_0$	30	^58^
Molecular weight (*g*/*mol*)	$D_{MW}$	914.2	^48^

The velocity-vector after angioplasty, obtained by solving the Eqs. (1 and 2), is displayed in Fig. 2c. A complex pattern of the velocity-field is observed conforming to the irregularity of the domain. The convective velocity components are believed to influence the distribution and retention of drug eluted from a balloon. During DCB angioplasty, the sirolimus release profile attains a quasi-steady state after 40 sec. which is in line with previous analytical as well as in-vitro studies (cf. Fig. 2d)^58^.

###  Model validation

**Figure 3:**
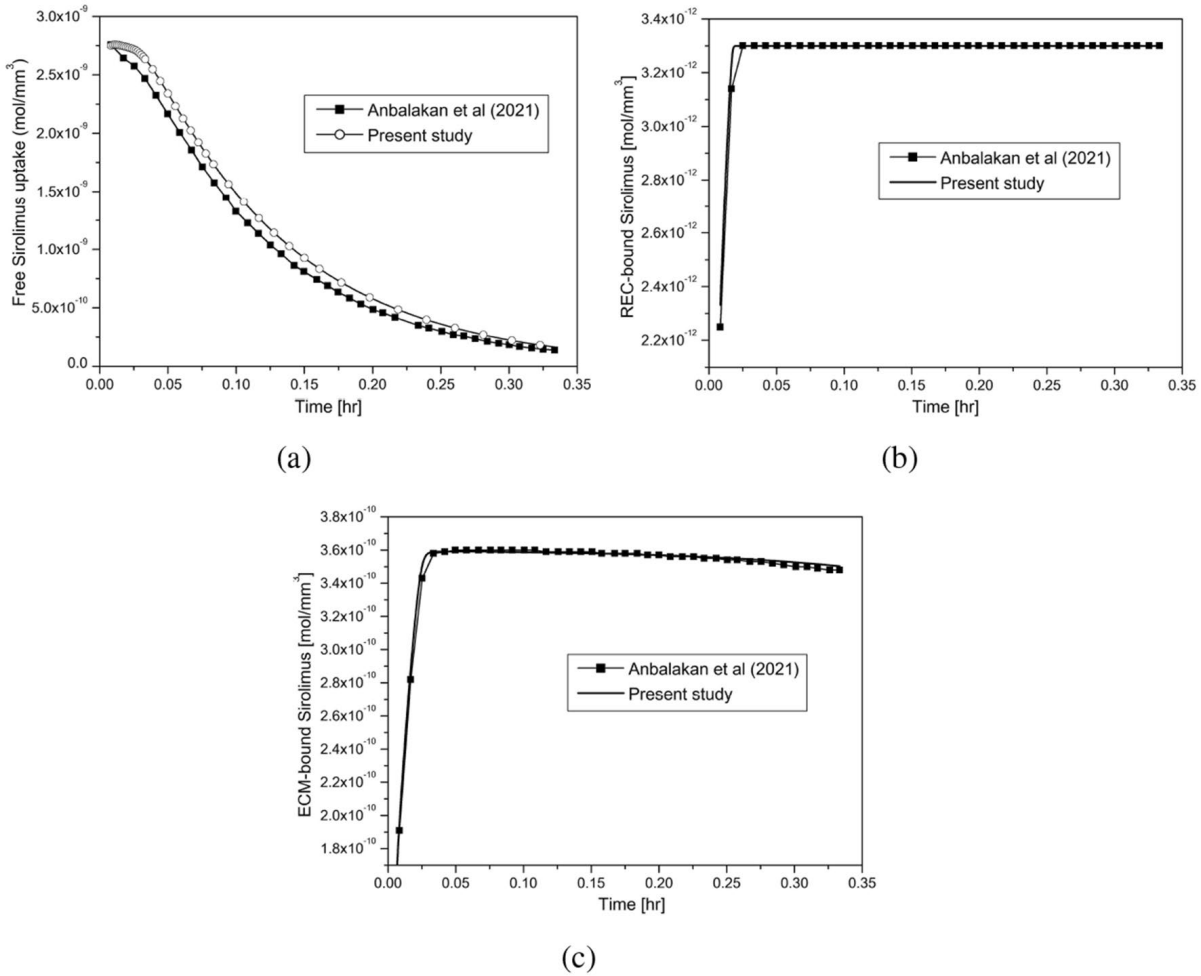
Model validation (Balloon inflation time = 30 s.): (**a**) Free sirolimus; (**b**) REC-bound sirolimus; (**c**) ECM-bound sirolimus.

A few comparisons with the findings of^58^ for the distribution of free, REC- and ECM-bound sirolimus, are shown in Fig. 3a–c. For this purpose, simulations have been carried out on a circular cross-section of a healthy arterial vessel (cf. Fig. 1d) with a distinction that the Brinkman Eqs. (1, 2) to represent interstitial flow through porous media in which pressure is calculated from the pressure equation (Section “Computational procedures”) has been made use of in this investigation, whereas the Darcy equation with a specified pressure gradient is considered in^58^. The amount of sirolimus uptake in our model and that of in^58^ are equal at 30 s, but they differ to a small extent at the beginning of the time period under consideration, despite the fact that their qualitative nature is the same. The reason for this difference can be attributed to the difference in considering the momentum transport equations in porous tissue (Brinkman vs. Darcy) and the pressure gradient (cf. Fig. 3a). The initial free drug concentration does decrease by 36.93% within 5 min, and 93.96% at 20 min. The saturation of REC- and ECM-bound sirolimus over time is shown in Fig. 3b,c, respectively. Also in this case, there is a very strong agreement. Hence, one may infer that the results obtained are unmistakably validated by the high degree of agreement.

### Influence of convection and lumen-tissue interface conditions

**Figure 4:**
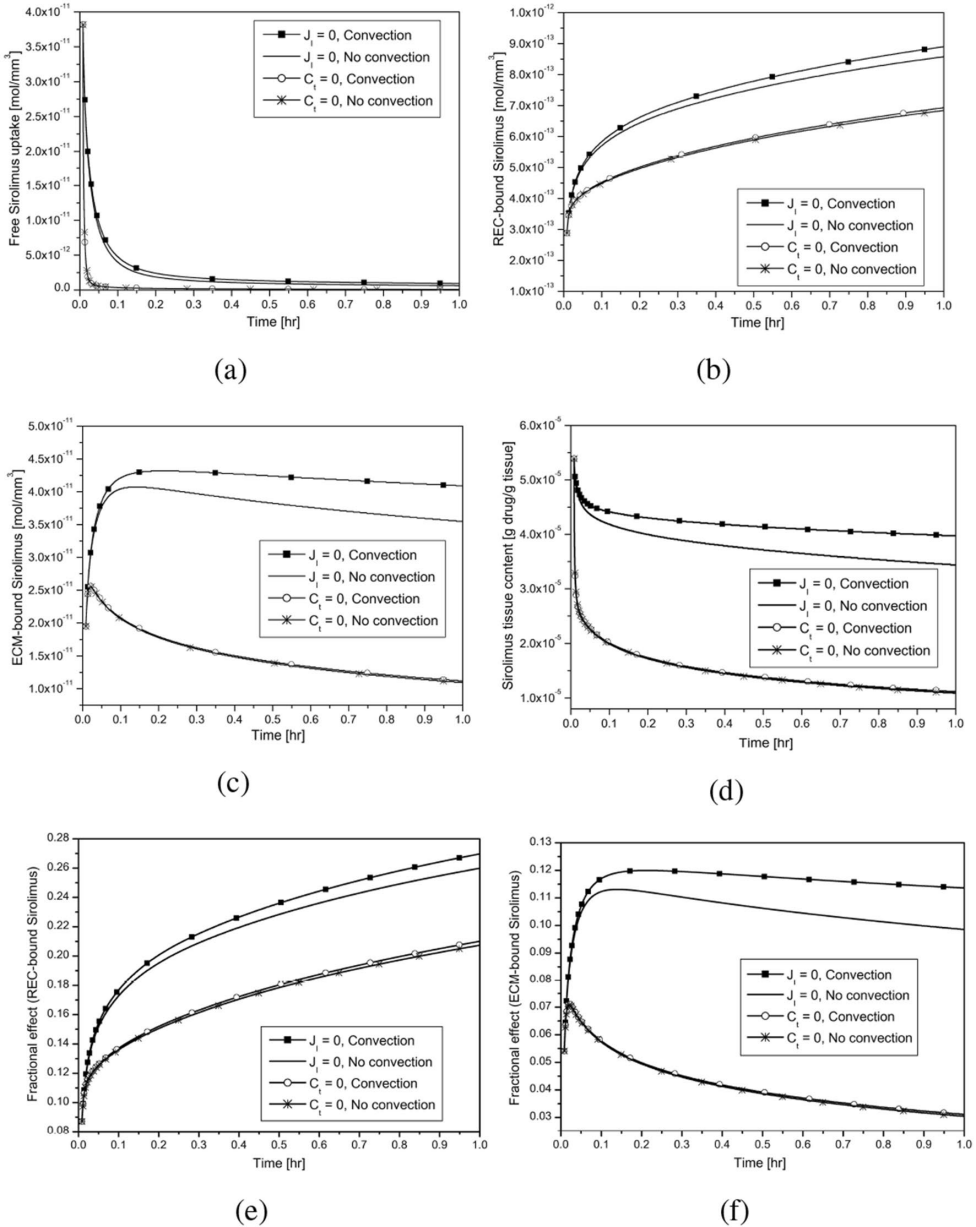
Effect of convection and interface condition (Ref. model; Balloon inflation time = 30 s): (**a**, **b**, **c**) Averaged drug concentration; (**d**) Tissue content; (**e**, **f**) Fractional Effect.

**Figure 5:**
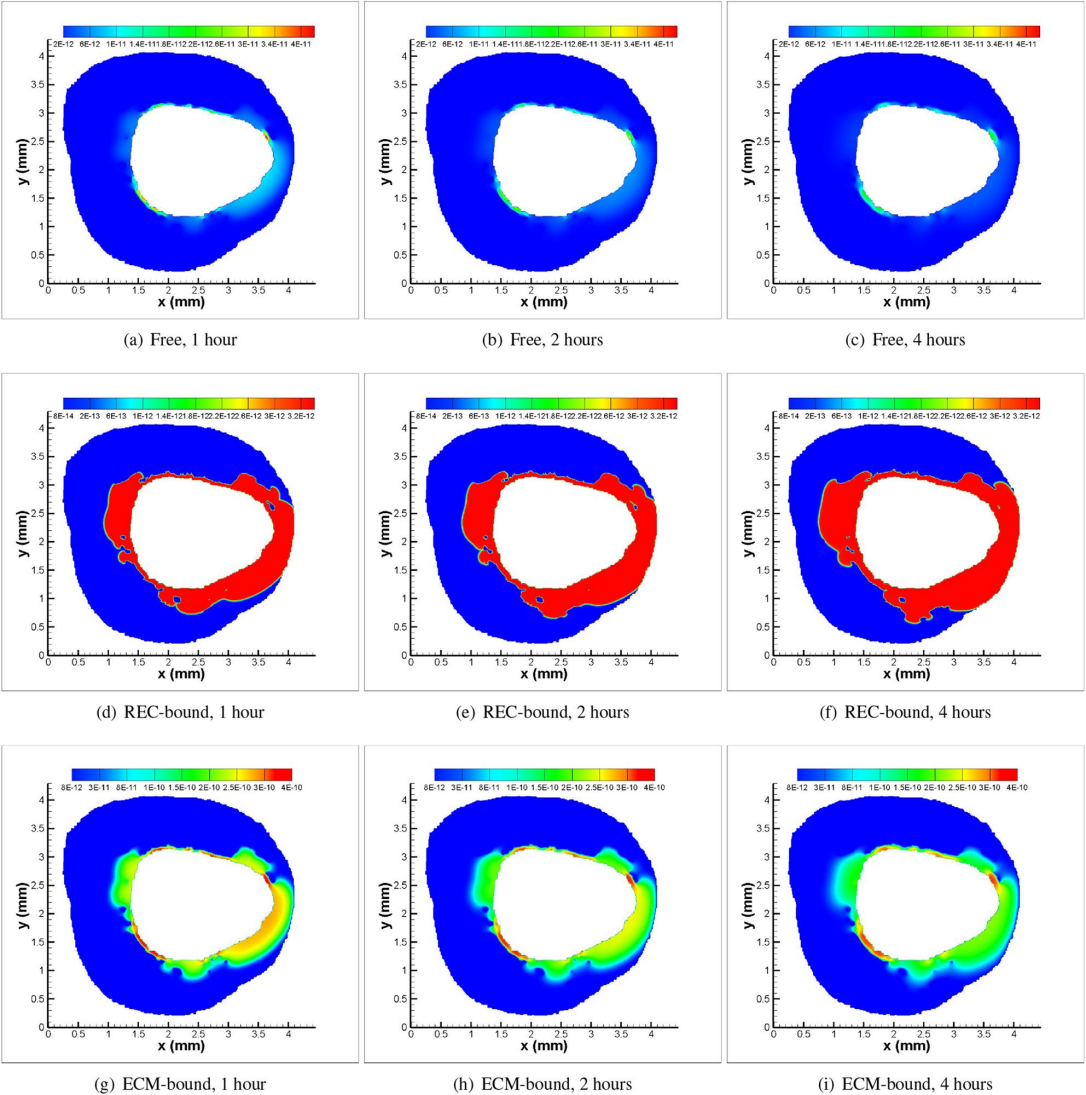
Spatio-temporal patterns for the Ref. model in the presence of convection and no-flux interface condition (Balloon inflation time = 30 s): (**a**, **b**, **c**) Free sirolimus; (**d**, **e**, **f**) REC-bound sirolimus; (**g**, **h**, **i**) ECM-bound sirolimus.

The impacts of lumen-tissue interface conditions (no-flux ($J_I=0$) and sink ($c_t=0$)) and the presence of interstitial fluid (convection) on the temporal variations of drug distribution and retention are investigated in an effort to determine how they affect DCB therapy, and the results are displayed in Fig. 4a,f. The averaged free sirolimus concentration follows a biphasic kinetics pattern, with a first-order decline phase followed by a slower efflux of free drug (cf. Fig. 4a). The clearance of the free drug is delayed for the zero-flux interface condition ($J_I = 0$) compared to the sink interface condition (Eq. 9, $c_t = 0$). Simulations predict that non-uniform interstitial velocity components (cf. Fig. 2c) cause the averaged concentration of free sirolimus to be long-lived, and the process eventually keeps more drug available for binding (cf. Fig. 4b,c). These results are consistent with previous findings when an atherosclerotic vessel with non-uniform plaque components was used to investigate DCB therapy^26^. Unlike our findings for healthy circular arteries, where binding site saturation occurs within 2 min (cf. Fig. 3b,c), the results in Fig. 4b,c fail to achieve binding site saturation within 1 h. According to simulations, REC-bound drug accumulates over time, with only 27% of binding sites occupied after 1 h in the presence of convection and no-flux interface condition (cf. Fig. 4b,e). In Fig. 4c, an initial build-up of ECM-bound drug is visible, followed by a sharp decline at t = 3 min in the case of sink interface condition, whereas the rate of decline slows and the peak concentrations shift to later times in the case of no-flux interface condition (cf. Fig. 4c,f). The tissue content (Eq. 11), which is defined as the total concentration left in the tissue per unit tissue weight, should be relevant to address in order to quantify the physiological characteristics of DCB delivery (cf. Fig. 4d). The presence of convection at a fixed time magnifies the tissue content, as expected and this effect is more pronounced for no-flux interface condition. Since convection results in the rapid influx of drug molecules, which eventually leads to a higher concentration of drug molecules and provides a higher tissue content. To manage drug transfer in the affected tissue, it is critical to understand how much drug is available for binding in the tissue; the fraction of bound drug (Eqs. 12, 13) is shown in Fig. 4e,f. The fraction of REC-bound drug increases with time, and convection and no-flux interface condition both promote rapid binding of specific receptors (cf. Fig. 4e). For sink interface condition, the fraction of ECM-bound drug increases initially before falling sharply, and the effect of convection is minimal (cf. Fig. 4f). However, for the no-flux interface condition, this fraction attains a peak and then gradually declines over time. In contrast to our findings in Fig. 4e, the fraction of ECM-bound drug in the Ref. model decreases over time.

Distribution patterns are significantly heterogeneous when variable diffusion coefficients associated with plaque compositions are used to model drug transport. The area under concentration (AUC) and peak concentration for free sirolimus decrease as time progresses from 1 to 4 h (cf. Fig. 5a–c), but increase for REC-bound sirolimus (cf. Fig. 5d–f). On the contrary, the AUC and the peak concentration for ECM-bound drug do decrease with increasing time from 1 to 4 h. The patterns depicted in Fig. 5 show the heterogeneous distributions of all drug forms owing to the heterogeneity of the plaque components, confirming their non-negligible impact in endovascular therapies using DCB. Predicted results show lower drug concentration within the pockets of calcified and necrotic cores owing to low drug diffusivity within these pockets.

### Influence of varying tissue compositions

**Figure 6:**
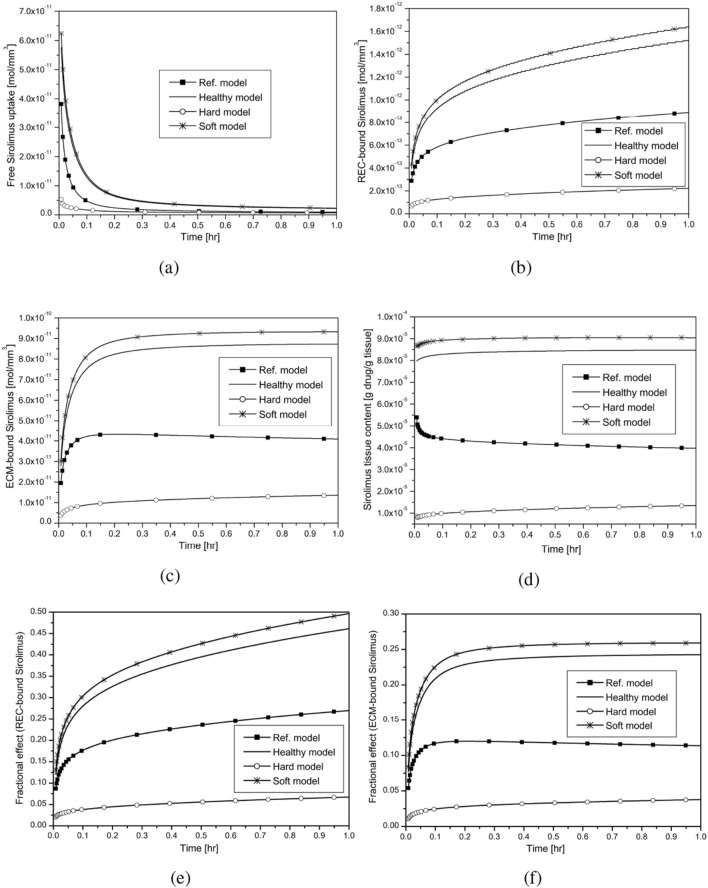
Effect of varying tissue compositions in the presence of convection and no-flux interface condition ($J_I = 0$, Balloon inflation time = 30 s): (**a**, **b**, **c**) Averaged drug concentration; (**d**) Tissue content; (**e**, **f**) Fractional effect.

**Figure 7:**
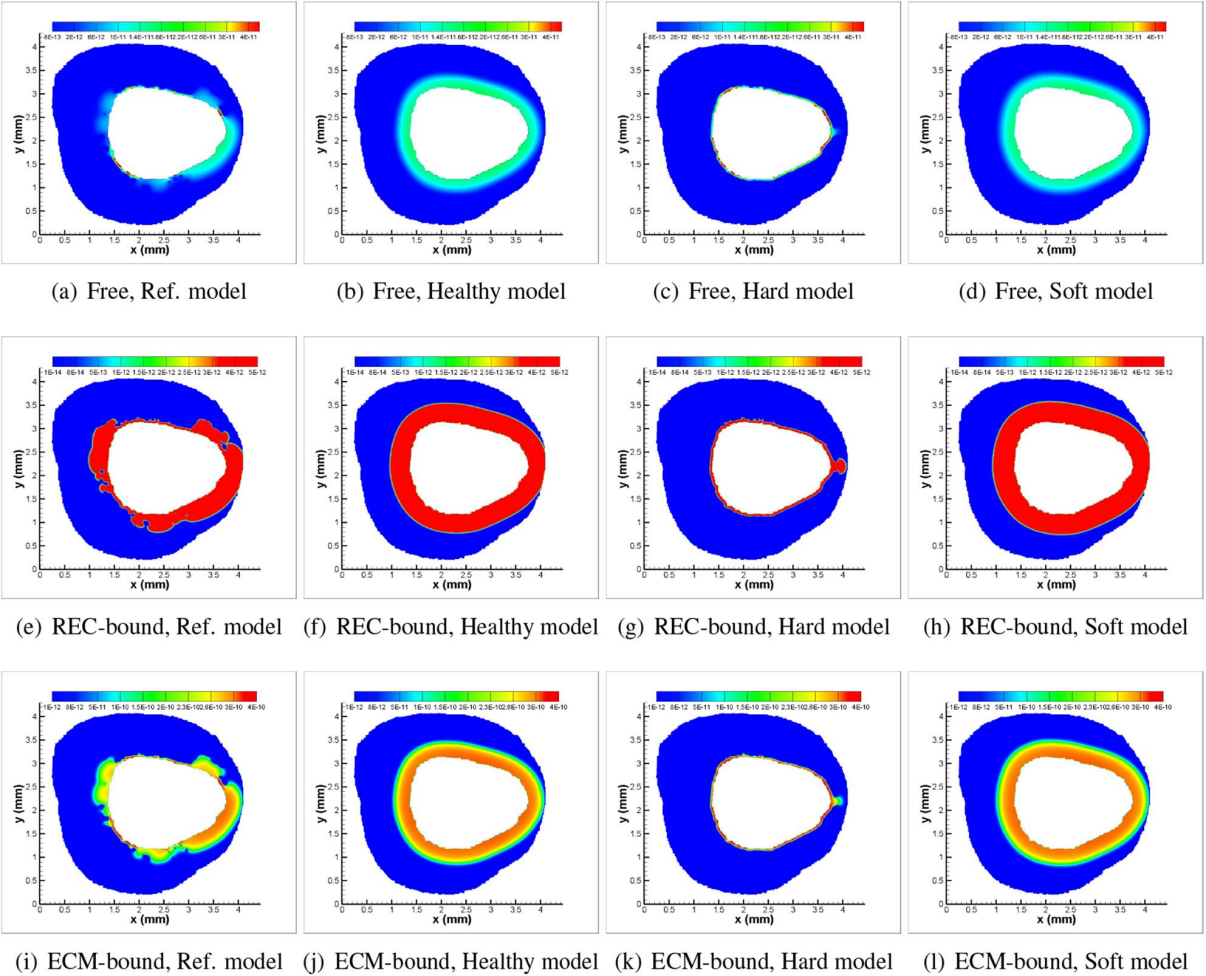
Spatial patterns for varying tissue compositions in the presence of convection and no-flux interface condition ($J_I = 0$) at $t=30$ min. (Balloon inflation time=30 s): (**a**, **b**, **c**, **d**) Free; (**e**, **f**, **g**, **h**) REC-bound; (**i**, **j**, **k**, **l**) ECM-bound sirolimus.

As arterial ultrastructure modulates endovascular delivery, we aim to study the influence of varying tissue compositions on DCB therapy using four different geometries, namely, (i) Ref. model (cf. Fig. 1a), (ii) Hard model (cf. Fig. 1b), (iii) Soft model (cf. Fig. 1c) and (iv) Healthy model (cf. Table 1). Recall that our Ref. model consists of 27.4% HT, 31.9% FI, 9.2% FF, 22.4% NC, and 9.1% DC; the hard model contains 27.4% HT and 72.6% NC & DC; the soft model is made up of 27.4% HT and 72.6% FI & FC and the healthy model consists of 100% HT. Fig. 6a–c display how the concentrations of free, REC- and ECM-bound sirolimus are perturbed over time for varying tissue compositions. It is worthwhile to note that sirolimus has 10-fold enhanced diffusivity in regions of FI and FF ($\mathcal {O}{10^{-4}}$) and a 100-fold lower diffusivity in the DC and NC regions ($\mathcal {O}{10^{-7}}$) than that in the healthy tissue ($\mathcal {O}{10^{-5}}$) (cf. Table 2). At 30 s, the soft model experiences maximum uptake of free sirolimus from the inflated balloon (cf. Fig. 6a), and the hard model’s averaged free sirolimus concentration is always lower than that of the other models considered. In the soft model, the distribution of free sirolimus is more evenly distributed (cf. Fig. 6a). This observation may be rationalised in the sense that the averaged concentration increases with the tissue’s area of increased diffusivity. The concentrations of REC- and ECM-bound sirolimus are all-time higher in the soft model due to high drug diffusivity within the FI and FF regions (cf. Fig. 6b,c). Unlike in Fig. 3b,c, the saturation of binding sites for both REC and ECM bindings is delayed and does not occur until $t = 1$ h. One intriguing feature to be noted is the concentration of REC-bound sirolimus increases with the passage of time under consideration, but that of ECM-bound sirolimus attains a quasi-steady state except for the hard model. In the hard model, the regions of DC and NC are limited to the lumen-tissue interface, and due to diffusive hindrance as well as slow diffusion within these regions, the retention of drug is prolonged, which eventually increases both bound drug forms with time. Another interesting finding is that, for the healthy and soft models, the tissue content initially increases before reaching a quasi-steady state; however, for the Ref. model, the tissue content initially decreases before reaching a stationary state. The tissue content of the hard model increases over time (cf. Fig. 6d). Our observations are in excellent qualitative agreement with those of^24^ who studied the dissolution of PTX and its retention in the case of DCB delivery. The soft model has a receptor occupancy of $\sim$ 50% in 1 h, which is $\sim$ 22% higher than the Ref. model (cf. Fig. 6e,f). The hard model, on the other hand, has only $\sim$ 7% receptor occupancy, which is $\sim$ 20% lower than the Ref. model. Another intriguing phenomenon is that the saturation of ECM binding sites decreases slowly over time for the Ref. model while it increases for the healthy, soft, and hard models (cf. Fig. 6f). However, the Ref. model experiences a $\sim$ 50% saturation of REC binding sites (not shown) and a $\sim$ 22% saturation in the hard model at 24 h, whereas the soft model experiences a $\sim$ 100% saturation in 20 hours for no-flux interface condition in the presence of convection (not shown). In the Ref. model, the ECM-bound drug reaches a quasi-steady state after 2 hours, and only $\sim$ 11% saturation occurs by 24 hours. While the ECM-bound sirolimus saturation in the hard model increases with time and reaches $\sim$ 13% at 24 hours (not shown), the ECM-bound saturation in the soft model drops over time and a $\sim$ 20% saturation is recorded at that time in the presence of no-flux interface condition and convection. Owing to impenetrable structural diffusion barrier in the hard and Type-A models, only large-time simulations for Type-A and hard models are appended in Sec. 3.5. Analysing all the results, one may conclude that the hindered diffusivity in the DC and NC regions prompts delayed saturation of both binding sites.

Drug distribution patterns are less differential when the same values of diffusivity are assumed for all five plaque components, which are equal to those for healthy tissue (cf. Fig. 7b,f,j). The AUC for free sirolimus is higher for the soft model (Fig. 7d) and it is least for the hard model (Fig. 7c) at $t=30$ mins in the presence of convection and no-flux interface condition. Because free sirolimus is minimally penetrable in the DC and NC regions, the AUC of free, REC- and ECM-bound sirolimus is minimal for the hard model. The computational results shown in Fig. 7a–l highlight the importance of accounting for lesion-specific transport properties that represent real-world scenarios. Furthermore, it is established that the regions of DC and NC confined to the interface pose a clinical threat to interventionalists, and pre-procedural treatment, like orbital atherectomy, may be needed prior to intervention to enhance the diffusivity in DC and NC regions.

### Influence of spatial distribution of plaque components

**Table 3:** Models with non-clustered pixels for each plaque component.

Model	Ref. model	Type-I	Type-II	Type-III	Type-IV
HT (%)	27.4	37	37	37	37
FI (%)	31.9	22.4	22.4	FI positions in Type-I are occupied by NC and vice-versa, 22.4 (NC)	FI positions in Type-I are occupied by NC and vice-versa, 22.4 (NC)
FF (%)	9.2	9.1	FF positions in Type-I are occupied by DC and vice-versa, 9.1(DC)	9.1	FF positions in Type-I are occupied by DC and vice-versa, 9.1 (DC)
NC (%)	22.4	22.4	22.4	22.4 (FI)	22.4 (FI)
DC (%)	9.1	9.1	9.1(FF)	9.1	9.1 (FF)
Geometry Considered					
Remarks		FI=NC, FF=DC, HT=37%	FF & DC positions are interchanged	FI and NC positions are interchanged	(FI,FF) and (NC,DC) positions are interchanged
RMSND (mm)	0.558	0.558	0.547	0.528	0.517

**Figure 8:**
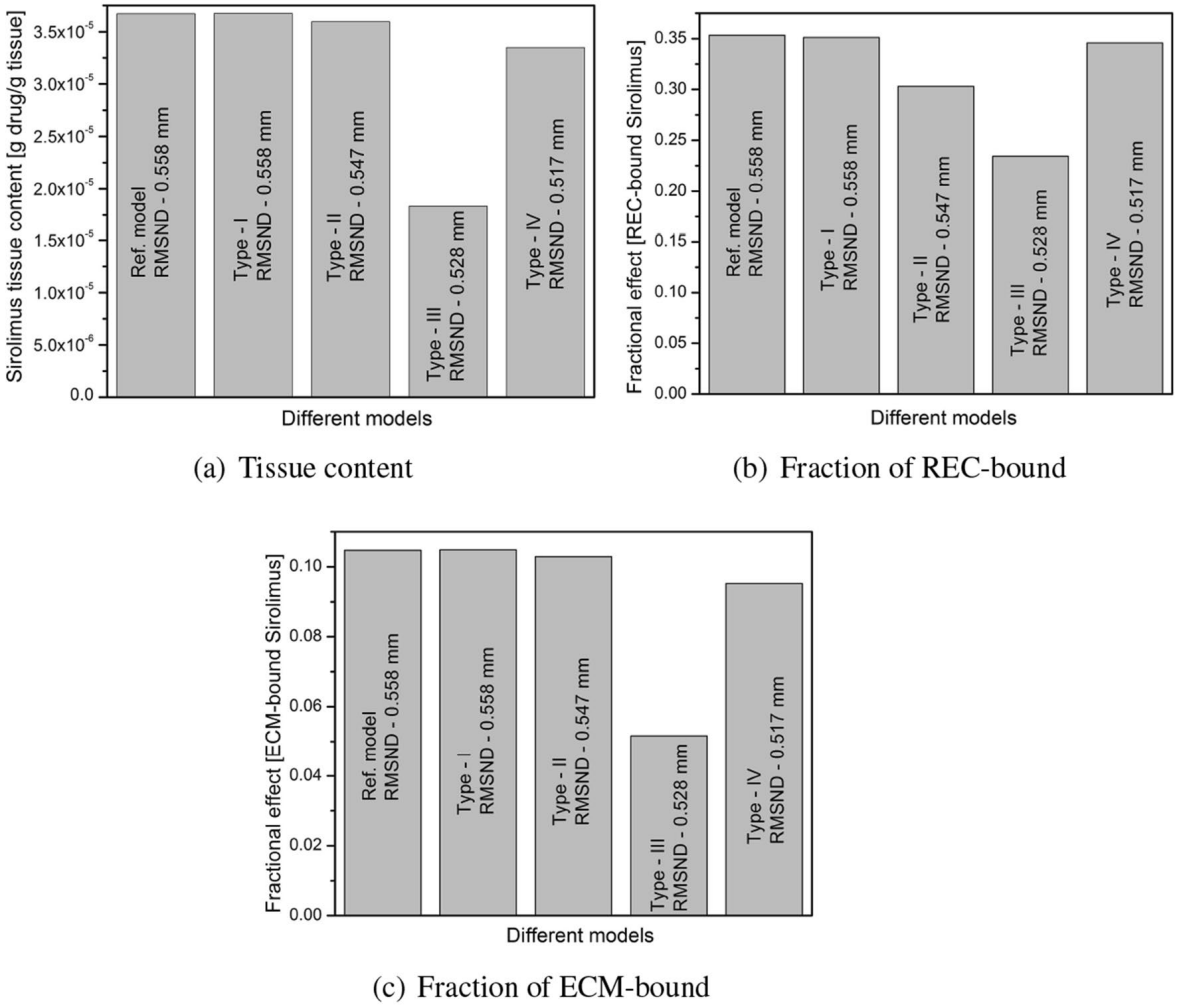
Effect of spatial positions of plaque components appeared in non-clustered manner at $t=4$ h. on (**a**) Tissue content; (**b**) Fraction of REC-bound sirolimus; (**c**) Fraction of ECM-bound sirolimus.

**Figure 9:**
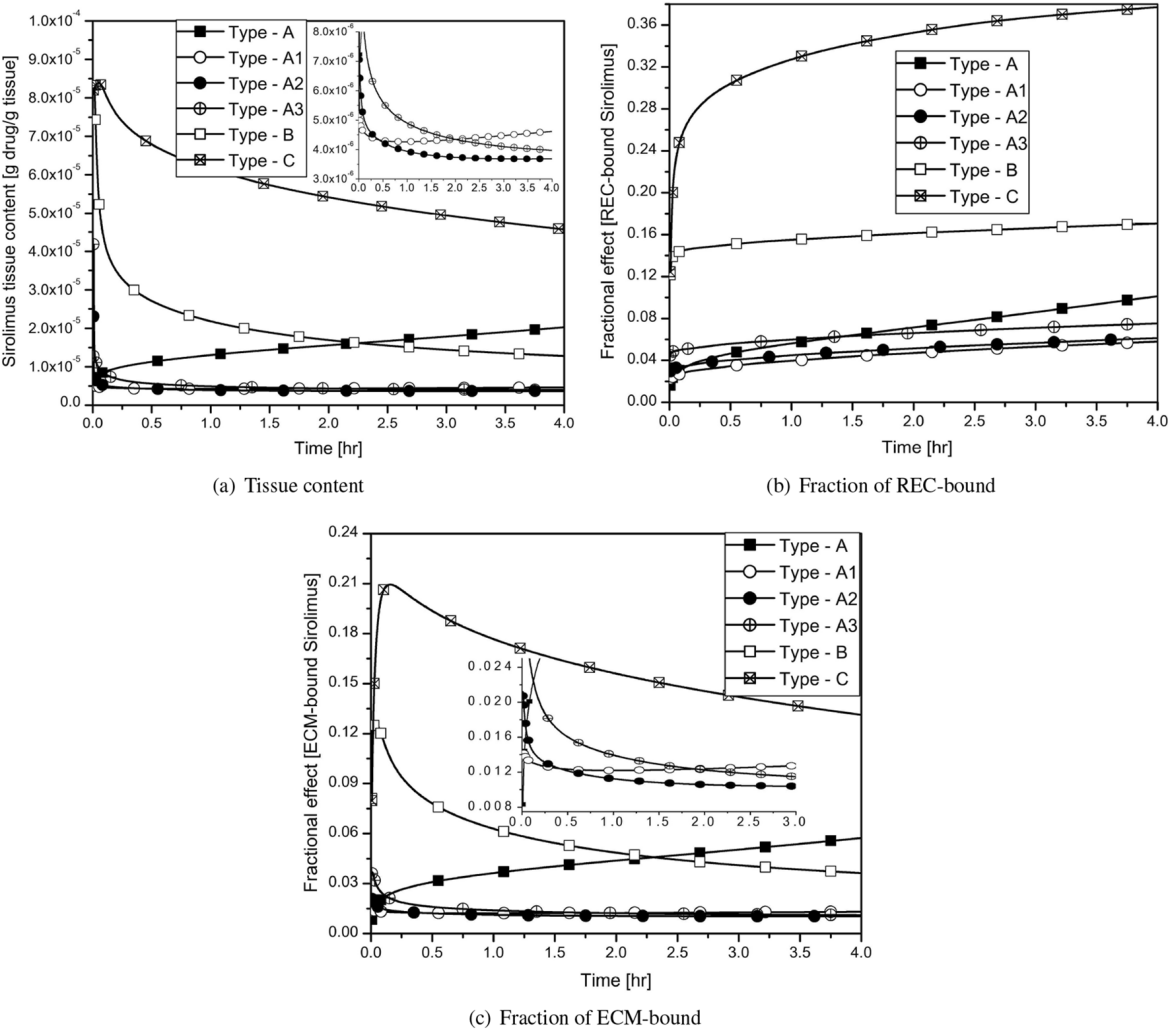
Effect of spatial positions of plaque components appeared in the clustered manner at $t=4$ hrs. on (**a**) Tissue content; (**b**) Fraction of REC-bound sirolimus; (**c**) Fraction of ECM-bound sirolimus.

We intend to investigate how the distribution of sirolimus eluted from a DCB is affected by changes in the spatial positions of the plaque components in relation to the lumen-tissue interface as well as their appearances in clustered or non-clustered forms. To the best of the authors’ knowledge, the dependence of drug distribution and retention on the spatial position of the individual plaque component in a realistic atherosclerotic domain is still unexplored, or at least, underexplored. More particularly, how do drug concentrations alter in response to changes in the positioning of the components in the therapeutic domain, while maintaining the percentage of occupancy of each plaque component as an invariant? To address this issue, we have made further modifications to our Ref. model. The occupancy of FI and NC is kept constant at 22.4%, and those of FF and DC at 9.1%, so that the spatial positions of FI and NC, and FF and DC are easily interchangeable. We now reconstruct four different types of geometry (Type-I,II,III,IV) by altering the positions of the plaque components (cf. Table 3). We next calculate the root mean square normal distance (RMSND) for all DC and NC pixels from the lumen-tissue interface, showing that the closer the DC and NC components are to the interface, the smaller the values of RMSND. Figure 8a,b,c depict the dependence of tissue content, the fraction of REC- and ECM-bound sirolimus with the RMSND at $t= 4$ h. Predicted outcomes demonstrate that tissue content reduces when RMSND falls (from 0.558 to 0.528 mm). On the other hand, a further decrease in RMSND (0.517 mm; Type-IV) (that is, DC and NC components are closer to the interface) leads to an increase in the tissue content of sirolimus. The fractions of REC- and ECM-bound sirolimus exhibit analogous behaviour to that of the tissue content. An increase in tissue content in the Type-IV model is due to a larger portion of the luminal interface being surrounded by the components of DC and NC in the Type-IV model as compared to the Type-III models at that specific instant. The DC and NC regions limited to the luminal surface affect the input flux and the drug is long-lived there due to slower diffusion. Thus, we may infer that the tissue content and fractions of all bound drug forms are linearly proportional to the RMSND up to a certain threshold distance at $t=4$ h, beyond that, they are inversely proportional provided the plaque components are scatteredly (non-clustered) distributed. However, the tissue content decreases with decreasing RMSND for all types (I–IV) at smaller times (not shown). To strengthen our justification further, some models of idealised atherosclerotic vessels are exhibited in Fig. 9 where the pixels of DC and NC components are grouped in clusters.

**Table 4:** Idealised models with clustered pixels for each plaque component.

Model	Type-A	Type-A1	Type-A2	Type-A3	Type-B	Type-C
HT (%)	37	do	do	do	do	do
FI & FF (%)	44.8	do	do	do	do	do
DC & NC (%)	18.2	do	do	do	do	do
Geometry Considered		Not shown	Not shown	Not shown		
Remarks	NC & DC positions are limited to the interface only	NC & DC positions are limited to one pixel ($8.48 \times 10^{-4}$ cm) away from the interface	NC & DC positions are limited to two pixel ($2 \times 8.48 \times 10^{-4}$ cm) away from the interface	NC & DC positions are limited to Four pixel ($4 \times 8.48 \times 10^{-4}$ cm) away from the interface	NC & DC positions are further away from the interface than Type-A3	NC & DC positions are further away from the interface than Type-B
RMSND (mm)	0.115	0.132	0.141	0.155	0.248	0.387

Our objective is to ascertain what transpires when the various components of a plaque are dispersed in various manners. In this study, we idealise the therapeutic domain as comprising of HT (37%), FI and FF (44.8%), DC and NC (18.2%). First, we assume that the regions of DC and NC are solely on the luminal surface (Type-A). Subsequently, the DC and NC regions are shifted one (Type-A1), two (Type-A2), and four (Type-A3) pixels (one-pixel length = $8.48 \times 10^{-4}$ cm) away from the interface, while the regions of FI, FF, and HT occupy the luminal surface area (cf. Table 4). To obtain Type-B geometry, the DC and NC regions are further moved away from the interface and finally, by moving the DC and NC regions away from the interface than Type-B and towards the perivascular end, we finally get Type-C. The regions of DC and NC are unable to completely cover the domain’s cross-section in Type-C (cf. Table 4). It’s interesting to notice that the tissue content and fractional effects of the two-phase bound drug do grow with time if the DC and NC regions are clustered around the interface and completely cover it (cf. Fig. 9a–c; Type-A). When the DC and NC regions are clustered together but one pixel far from the interface, the tissue content and ECM-bound drug concentration first decline, then increase (cf. Fig. 9a,c, magnified insets; Type-A1), whereas the concentration of REC-bound drug steadily increases (cf. Fig. 9b). When the DC and NC regions are two and four pixels distant from the interface, the tissue content drops from a higher value (cf. Fig. 9a, magnified inset; Types-A2,A3). The tissue content for Type-B shows a sharp decline as time progresses, whereas it is higher for a short period of time initially, followed by a relatively slower decline in Type-C. The multiple cross-overs between curves for various types emphasise the time-dependent nature of outcomes (cf. Fig. 9a,b,c). However, at $t=4$ h, the tissue content decreases with decreasing RMSND (from 0.387 to 0.141 mm) except for Types-A1 where it increases with the decrease in RMSND (from 0.141 to 0.115 mm). Thus, we may infer that when the DC and NC regions are restricted to the luminal interface (Type-A) or extremely close to it ( one pixel away from it, Type-A1) in clustered form, the tissue content is inversely proportional to the RMSND, and directly proportional otherwise. Here, the Type-A2 geometry (two pixels from the interface) serves as the threshold for two separate proportionalities. The fraction of REC-bound drug decreases as RMSND increases for Types-A and A1, but it increases for other types (cf. Fig. 9b). The drug fraction bound to the ECM behaves similarly to the tissue content (cf. Fig. 9a,c). Thus, one comes to the conclusion that in atherosclerotic vessels, the plaque components and their positioning in the therapeutic domain play a significant role in DCB delivery performance. It may further be inferred that the regions of DC and NC limited to the interface or very close to it should be given special care prior to intervention.

### Large-time behaviour of DCB therapy in the hard and type-A models

**Figure 10:**
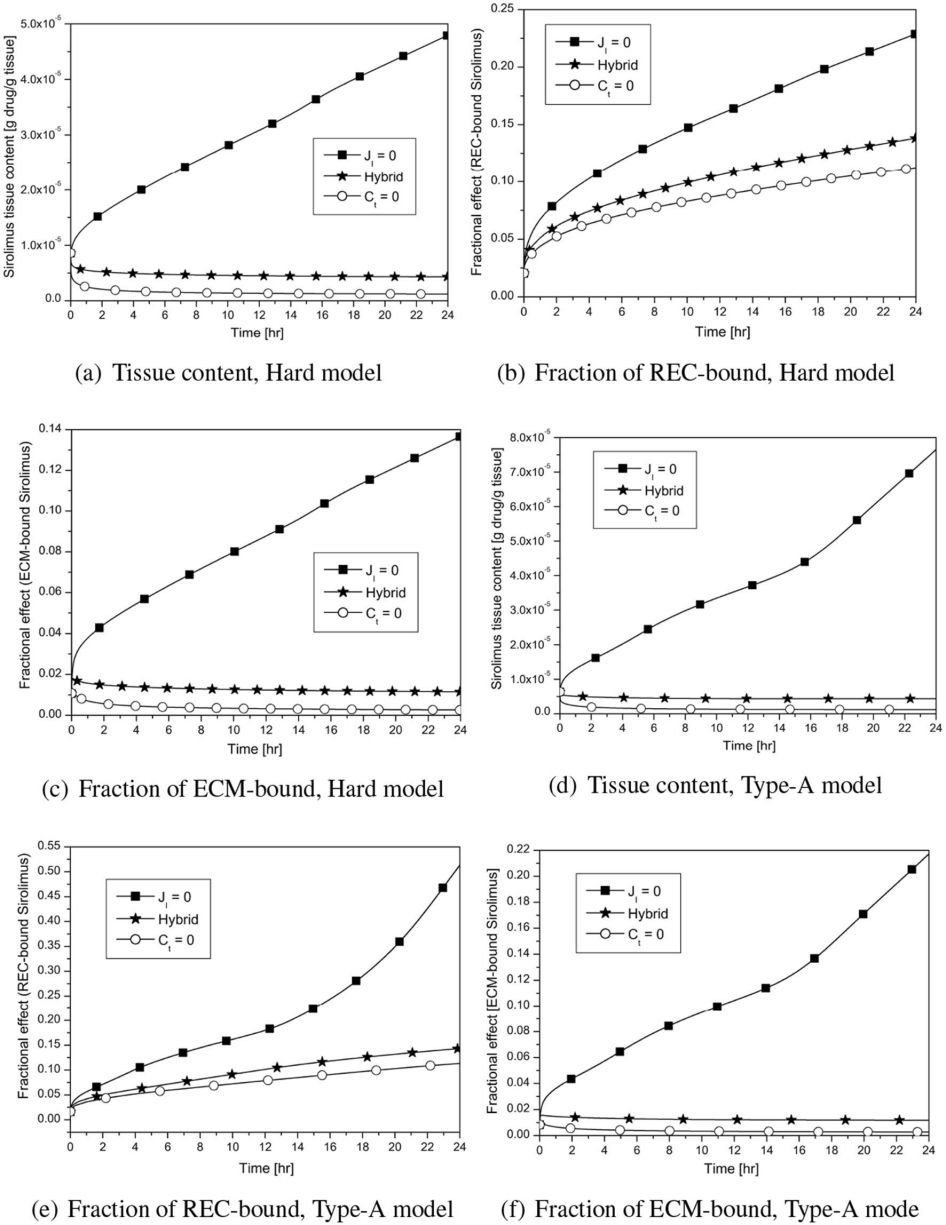
Large time behaviour of DCB therapy in hard and type-A models in the presence of convection. Balloon inflation time = 30 s): (**a**, **b**, **c**) Hard model; (**d**, **e**, **f**) Type-A model.

Diverse tissue compositions influence how drugs are distributed and retained in the blood vessels. The effectiveness of DCB therapy is also determined by how they appear and are positioned in relation to the interface. Here, we’ll demonstrate how the DCB therapy behaves differently for shorter and longer periods of time when applied to hard and Type-A models. In order to keep this short, only the long-term behaviour of tissue content and the fractions of REC- and ECM-bound sirolimus for various interface conditions in the hard and Type-A models is shown in Fig. 10a–f. It should be noted that there are no qualitative changes in the tissue content and the fractions of REC- and ECM-bound sirolimus with time in the Ref. and soft models. The DC and NC regions are non-clustered in the hard model (which only includes HT, DC, and NC in non-clustered form; cf. Fig. 1b), whereas in the Type-A model, they appear in clustered form and are restricted to and entirely cover the luminal interface (cf. Table 4). The hard model’s initial build-up of tissue content and the fraction of sirolimus that is bound to the ECM do grow with time, and at roughly 18 h, there is a cross-over between the hard model’s curves and the Ref. Model’s curves (not shown). But there, the fraction of sirolimus bound to REC is at an all-time low (not shown). However, the tissue content and both the fractions of sirolimus bound to the ECM and REC in the Type-A model do rise with time, and at 24 h, their values are larger than those in the other models (Types B, C) (not shown). One may draw the conclusion that the hard model and the Type-A model act in different ways over time by comparing the results of Figs. 6, 9 and 10.

We review how interface conditions affect the hard and Type-A models to address the escalation of these factors. The tissue content and the fraction of drug that is bound to the ECM in the hard model both decrease from their respective peak values after the balloon deflates and reach their respective quasi-steady states in the case of the sink interface condition; however, following^26^, their values progressively rise as the sink condition approaches the no-flux via the hybrid (in between no-flux and sink conditions) interface condition. A greater value results from the no-flux interface scenario, and it is higher for the entire time period taken into account. However, for all interface conditions taken into account, the fraction of drug that is REC-bound steadily increases with time. In the case of the Type-A model, an analogous behaviour for various interface conditions is noted. In the hard model, a $\sim$ 22% REC-saturation by 24 h is noted, whereas it is above 50% in the Type-A model by this time for no-flux interface condition in the presence of convection. The ECM-bound sirolimus saturation in the hard model increases with time and reaches $\sim$ 13% at 24 h which is $\sim$ 21% in the Type-A model at that time in the presence of no-flux interface condition and convection. Thus, it can be concluded that the emergence of the DC and NC regions and the rough approximation of interface conditions by sink or no-flux considerably influence the efficacy of DCB therapy.

### Sensitivity analysis

**Figure 11:**
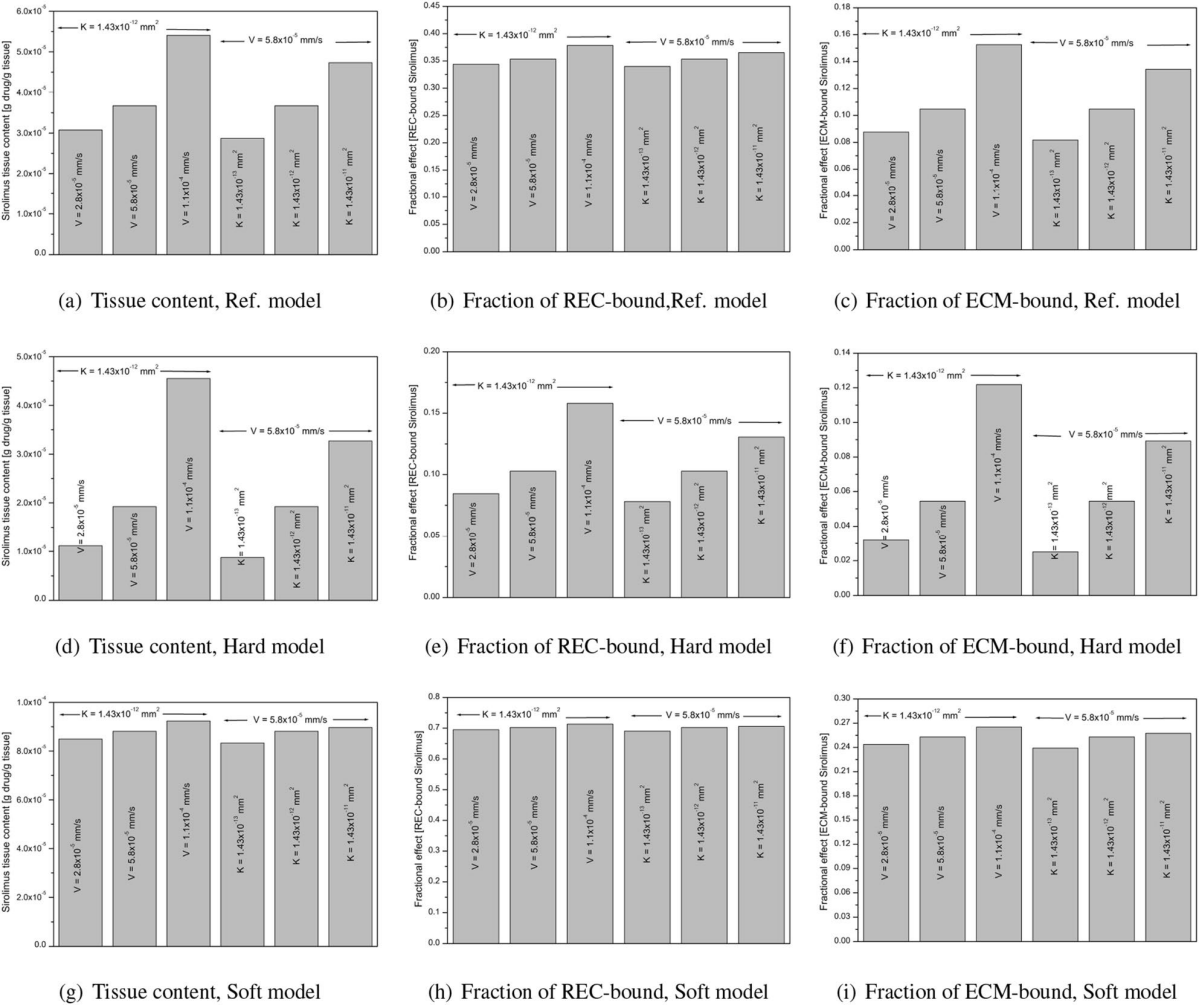
Sensitivity analysis at $t=4$ h.: (**a**, **d**, **g**) Tissue content; (**b**, **e**, **h**) Fraction of REC-bound sirolimus; (**c**, **f**, **i**) Fraction of ECM-bound sirolimus.

Because of the different experimental and theoretical modelling environments, as well as uncertainties in the parameters involved, a thorough sensitivity analysis is performed to estimate the influence of the filtration velocity (*V*) and the tissue permeability (*K*) on the tissue content and the fraction of REC- and ECM-bound drug in the Ref., hard and soft models. Figure 11a–i displays how these pharmacological factors are perturbed by varying tissue compositions. Several studies demonstrated that the filtration velocity is of $\mathcal {O}{10^{-5}}$ mm/s^72–75^ and the permeability ranges from $\mathcal {O}{10^{-13}}$ mm^2^ to $\mathcal {O}{10^{-11}}$ mm^2^^74,76–78^. Keeping this in mind, the value of the filtration velocity is assumed from 2.8$\times 10^{-5}$ mm/s to 1.1 $\times 10^{-4}$ mm/sec and that of permeability ranges from $1.43 \times 10^{-13} \textrm{mm}^2$ to $1.43 \times 10^{-11} \textrm{mm}^2.$ Simulated results predict that there is a sharp change in tissue content ($\sim$ 15% decrease and $\sim$ 47% increase from that for baseline filtration velocity of $5.8 \times 10^{-5}$ mm/s when the tissue permeability is kept fixed at $1.43 \times 10^{-12} \textrm{mm}^2$, and $\sim$ 22% decrease and $\sim$ 28% increase from that for baseline permeability of $1.43 \times 10^{-12} \textrm{mm}^2$ when the filtration velocity is kept fixed at $5.8 \times 10^{-5}$ mm/s; cf. Fig. 11a) and in fraction of ECM-bound drug ($\sim$ 16% decrease and $\sim$ 45% increase from that for baseline filtration velocity of $5.8 \times 10^{-5}$ mm/s, and $\sim$ 22% decrease and $\sim$ 28% increase from that for baseline permeability of $1.43 \times 10^{-12} \textrm{mm}^2$; cf. Fig. 11c). In Ref. model, however, a mild change is noted in the fraction of REC-bound drug (only $\sim$ 2% decrease and $\sim$ 7% increase from that for baseline value for varying filtration velocity, and $\sim$ 2% decrease and $\sim$ 2% increase from that for baseline value for varying permeability; cf. Fig. 11b). On the contrary, the tissue content and the fraction of REC—and ECM-bound drug are sensitive to the filtration velocity and the tissue permeability in the hard model (cf. Fig. 11d–f). In this model, the respective decrease and increase of the tissue content are $\sim$ 41% and 136% for varying filtration velocity; $\sim$ 95% and $\sim$ 70% for varying permeability. The fraction of REC-bound drug experiences $\sim$ 18% decrease and 54% increase for varying filtration velocity, and $\sim$ 24% decrease and $\sim$ 70% increase for varying permeability. However, the respective percentages for the fraction of ECM-bound drug are ($\sim$ 41%, $\sim$ 123%) for varying filtration velocity and ($\sim$ 54%, $\sim$ 64%) for varying permeability. The soft model experiences the least perturbation, as depicted in Fig. 11g-i. Only $\sim$ 4% decrease and $\sim$ 3.8% increase for varying filtration velocity, and $\sim$ 4.2% decrease and $\sim$ 1.7% increase for varying permeability in the tissue content are recorded. The respective decrease and increase in the fraction of REC-bound drug for varying filtration velocity and varying permeability are ( $\sim$ 1%, $\sim$ 1.5%) and ($\sim$ 1.8%, $\sim$ 0.5%). The corresponding values for the fraction of ECM-bound drug are ( $\sim$ 1%, $\sim$ 4%) and ($\sim$ 5.5%, $\sim$ 1.8%). Analysing all the results, one can conclude that the hard model is more sensitive and the soft model is less sensitive to filtration velocity and permeability, implying that adequate knowledge of plaque compositions is necessary for the improved efficacy of DCB therapy.

## Discussion

In comparison to other therapies, the endovascular delivery via a drug-coated balloon is better because it does not require the use of permanent indwelling implants. We still have a lot to learn about the way antiproliferative drugs are delivered using balloon catheters as well as how well they work when the therapeutic domain contains a variety of plaque compositions. Clinical studies have not entirely demonstrated that they produce a long-lasting, persistent benefit^79^. In addition, our research suggests that the spatial arrangement of each plaque component and whether they seem clustered or not have a substantial impact on the endovascular delivery of the drug and its retention in the therapeutic domain. These trials can be guided by animal models and pre-clinical research, even though the majority of pre-clinical investigations to date have used models of healthy or idealised arterial vessels^24,32,79,80^. There are a few studies that focus on DCB therapy in a region with different tissue compositions^14,23,26,27,38,58^, but to the best of the authors’ knowledge, there is no such study that examines the impact of the spatial positions of plaque components and their appearance with respect to the lumen-tissue interface on endovascular drug delivery from coated balloon.

In this study, we aim to develop a computer model of drug transport in diseased arterial cross-sections, where the positions of plaque components relative to the interface have been given due consideration while maintaining their proportions. This investigation strongly agrees qualitatively as well as quantitatively with those of^58^ in a healthy circular model (cf. Fig. 3). Simulations predict early saturation of binding sites in a healthy circular domain as compared to other models (cf. Figs. 3, 6). Thus, the impact of non-uniform tissue compositions on DCB therapy can be put on record while optimising the efficacy of this device. Given the importance of two opposing interface conditions, our study predicts a negligible impact of convection on the tissue content and the REC- and ECM-bound drug in the case of the sink interface condition ($c_t=0$), while convection does amplify them for the no-flux interface condition ($J_I=0$)(cf. Fig. 4).

There are a number of intriguing findings obtained from this investigation. First, several models (Ref. model, hard model, soft model, and healthy model) have been taken into consideration to examine the impact of varying tissue compositions (cf. Figs. 1, 6, and Table 1). The concentration of sirolimus bound to REC and ECM is at an all-time high in the soft model due to 10-fold enhanced diffusivity in the FI and FF regions, but due to 100-fold decreased diffusivity in the DC and NC regions and their appearances being restricted to the interface, the hindered diffusivity in the DC and NC regions causes delayed saturation of binding sites. Second, keeping the proportion of each plaque component unaltered, our investigation predicts the effect of the spatial distribution of tissue compositions on sirolimus tissue content and the fraction of REC- and ECM-bound sirolimus (cf. Table 3, Fig. 8). Here, apart from the Ref. model, we construct four different types of model geometry (Types-I,II, III, and IV) by altering the positions of plaque components. Based on the root mean square normal distance (RMSND) for all DC and NC pixels from the interface, our investigation predicts that the tissue content and the fraction of both bound drug forms do decrease with decreasing root mean square normal distance (RMSND), except in the scenario when the DC and NC regions are quite close to the interface. This is because the regions of DC and NC in Type-IV impede the input flux, and the drug is long-lived therein. To put a strong note on this point, we recreate certain idealised models (Types-A, A1, A2, A3, B, and C) in which the plaque components are dispersed in a clustered fashion (cf. Table 4, Fig. 9). It is interesting to note that, as long as the DC and NC regions are grouped and restricted to the interface (cf. Fig. 9, Type-A), the tissue content and two-phase bound drug forms do increase over time. However, this phenomenon does change as the regions of DC and NC are shifted towards the perivascular end (Type-A1 $\rightarrow$ Type-C). Thus, one may infer that the clustered regions of DC and NC limited to the interface or very close to it should be given special care for the success of DCB therapy, as in these scenarios, short- and long-time behaviours of the tissue content and two-phase bound drug seem to be different, and hence, extensive VH-IVUS imaging is needed to detect the actual tissue compositions. Careful validation using bench-top experiments or animal studies may allow for an improved understanding of the efficacy of DCB therapy.

### Study limitation and future direction

This investigation deals with a specific cross-sectional image of an atherosclerotic artery (Ref. model) with heterogeneous plaque compositions. For the inferences derived in this paper to be generalisable, simulations of this nature need to be performed on other patient-specific geometries. Since one of our objectives is to study the impact of positional variations of each plaque component in non-clustered form on endovascular delivery using DCB, we have reconstructed a number of model geometries from the Ref. model (Types- I, II, III, and IV) (cf. Table 3). To strengthen our findings further, we recreated another set of model geometries (Types A, A1, A2, A3, B, and C) (cf. Table 4) based on the clustered position of DC and NC regions about the interface and obtained some fascinating results. Thus, a number of ten model geometries have been constructed from the specific geometry to look into the objective of this investigation.

Like any computational modelling study, our investigation is based on a number of parameter selection assumptions that we have made. Several parameters were collected from the literature and calculated through a series of bench-top or animal model tests. Although not perfect, this method of acquiring parameters from many sources enables us to pinpoint some understudied elements in determining the efficacy of DCB therapy in tissues with various compositions. Since the US Food and Drug Administration has issued a caution regarding the use of DCB, several diligent researchers have made some noteworthy additions to our understanding of the effectiveness of this device. Angioplastic pressure^32,81^, various payloads^82^, coating morphology^32,34^, the pattern of the balloon’s surface^33^, coating dissolution kinetics^24^, coating-endothelial bond failures^30^, etc. are some critical factors that have been studied in recent years. In addition, the investigation’s novel feature focuses on the spatially diverse tissue compositions and how they affect DCB therapy.

A concern about the application of DCB is the possibility of the slow-flow phenomenon^83^. It is believed that this resulted from particle embolisation with DCB application, which could potentially account for the worse outcomes with PTX DCB use as reported in the meta-analysis^11^. According to the analysis of angiographic images taken after drug elution, there is no evidence of a slow-flow phenomenon when using sirolimus DCB because of micro-reservoirs of phospholipid polymer complex with the cell adherent technology, which minimises distal embolisation (SAVE Trial 2022)^84^. In the line of^42,58^, we assume rapid dissolution of sirolimus. A noteworthy investigation into coating dissolution in the context of DCB therapy^24^ suggests that concerns about potentially late toxicity stemming from PTX’s slow dissolution from coating emboli are indicative of potential local toxicities. Additionally, a coating that dissolves slowly is exposed to luminal flow for a longer period of time. This eventually leads to a higher fluid shear-induced lysis of the coating, which could prove adverse to the body over an extended period of time. Incorporating all of these factors into one nest is an uphill task, despite the authors’ awareness that excluding any one of them could result in an under- or overestimation of the findings. However, an honest effort is made to look into several understudied factors that may be the cause of DCB’s limited usage in different plaque compositions. In our ongoing work, we want to go over these topics again.

### Conclusion

The diffusivity in the DC and NC regions is 100-fold less than that in the HT (cf. Table 2). The present investigation demands an intensive VH-IVUS scan to know precisely the composition of a plaque and the spatial position of each plaque component as well. The intricate interplay between plaque heterogeneity, the spatial locations of individual plaque components, and their relative positions with respect to the lumen-tissue interface in clustered or non-clustered forms, the interstitial flow, and the interface condition are the focus of this work. Our study predicts the strong dependence of the tissue content and occupied binding sites on the positions of the DC and NC regions, even though they comprise the same volume throughout. Our study also shows varied behaviour of the tissue content and fractional effects for different cases, like whether the DC and NC regions are clustered or not. This model study will undoubtedly assist in better understanding the DCB delivery scenario by taking into account the enormous biological complexity. To make DCB delivery more effective, the above factors should be carefully thought through.

Simulated results predict some preliminary conclusions as

- Both convection and no-flux interface conditions do amplify the total tissue content and the retention of sirolimus (cf. Fig. 4).
- Diffusive hindrance in DC and NC regions is responsible for delayed saturation of binding sites (cf. Fig. 6).
- With regard to the patient-specific artery, the tissue content and bound sirolimus decrease with decreasing RMSND, except in the case when a significant portion of the DC and NC regions are limited to the interface (cf. Fig. 8).
- If DC and NC are limited to the interface only or are extremely close to the interface, the tissue content and the concentration of bound drug do increase with the passage of time, however, a reverse trend is observed when they are shifted away from the interface, confirming the significant impact of the spatial position of individual plaque components (cf. Fig. 9).
- Clustering of plaque components (patient-specific vs. idealised) influences much of the distribution and retention of free sirolimus (cf. Fig. 9).
- In the Ref. model, the value of the ECM-bound sirolimus attains a peak followed by a slow decline with the advancement of time in the case of no-flux interface condition (cf. Figs. 4c,f, 5g,h,i), whereas the value progressively increases in the hard and Type-A models (cf. Fig. 10c,f).

### Translational perspective

Effectiveness of DCB therapy depends on a number of factors, including the type of coated drug, effective excipients, rate of pharmacokinetics, drug load, release kinetic profiles, and drug loss during the delivery process. To improve the therapeutic efficacy of DCBs, lesion complexity, and other pertinent parameters must be taken into account. In spite of the fact that PTX is still one of the most widely used coating drugs for preventing cell proliferation, sirolimus-coated balloon therapy has attracted more interest because it lessens the development of atherosclerotic plaque by preventing macrophage proliferation, lipid accumulation, and plaque formation during angiogenesis^38,58,85^. The major mechanism during drug release and uptake is believed to be diffusion. In order to predict the effectiveness of DCB therapy, it is also necessary to take into account how sirolimus interacts with different tissue compositions. In reality, the therapeutic domain is an atherosclerotic plaque with heterogeneous tissue compositions, which leads to uneven drug absorption.

This investigation demonstrates that a plaque with DC and NC regions may limit the number of available binding sites. Also, the spatial distribution of DC and NC components contributes much to the tissue content of sirolimus and its retention, with DC and NC regions far from the interface resulting in increased tissue content and retention as well. However, if the DC and NC areas are only limited to the interface, their values are slightly greater. Following a thorough analysis of the data, it is possible to draw the conclusion that the success of DCB therapy depends strongly on plaque types, which may necessitate treatment before intervention^23^.

## Data Availability

All data generated or analysed during this study are included in this published article. The raw data are provided by the corresponding author upon reasonable request.
